# Supplementary material for: The interactome of intact mitochondria by cross-linking mass spectrometry provides evidence for coexisting respiratory supercomplexes
Source: Mol Cell Proteomics. 2017 Dec 8;17(2):216–32. doi: 10.1074/mcp.RA117.000470 (PMC5795388; doi:10.1074/mcp.RA117.000470)
Supplement: Supplemental Data [file supp_RA117.000470_133922_0_supp_23978_fzffwf.zip › spectra_annotation/mito_spectra_annotation/71-1-2-1-11-1.pdf]

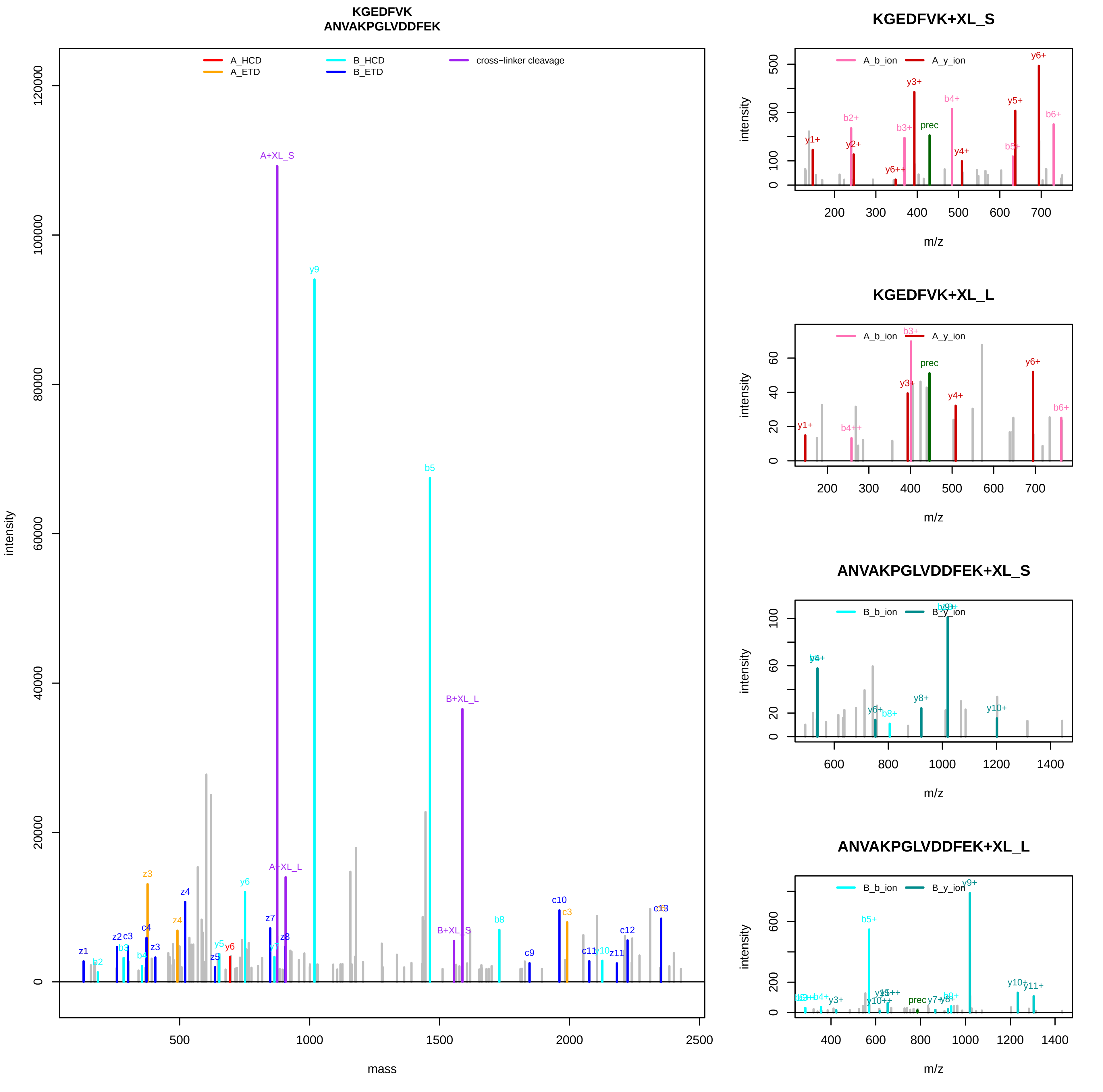
KGEDFVK
ANVAKPGLVDDFEK
A_HCD
A_ETD
B_HCD
B_ETD
cross−linker cleavage
A+XL_S
A+XL_L
B+XL_L
B+XL_S
y9
b5
y6
z3
z4
c4
c10
c3
c13
c12
c9
b8
intensity
mass
0
20000
40000
60000
80000
100000
120000
500
1000
1500
2000
2500
KGEDFVK+XL_S
KGEDFVK+XL_L
ANVAKPGLVDDFEK+XL_S
ANVAKPGLVDDFEK+XL_L
A_b_ion
A_y_ion
B_b_ion
B_y_ion
prec
y6+
y3+
b4+
y5+
b6+
b2+
b3+
y2+
y1+
y4+
b5+
y6++
b4++
y9+
y8+
y10+
b8+
y11+
m/z
